# Rainwater harvesting system installations required to offset new water demand created by growing populations in Broward and Palm Beach Counties: A dataset for decision making based on numbers of installations, costs, and water and energy savings

**DOI:** 10.1016/j.dib.2019.105016

**Published:** 2019-12-17

**Authors:** Kurt Wurthmann

**Affiliations:** Nova Southeastern University, USA

**Keywords:** Economic analysis, Energy costs, Energy intensity of water, Lawn irrigation, Rainwater harvesting, Southeast Florida, Water supply

## Abstract

This article presents yearly data for the period 2020–2060 on estimates for population growth and associated numbers of new and existing single-family detached homes to be fit or retrofit with rainwater harvesting (RWH) system equipment for lawn irrigation in order to continuously offset new water demand created by new residents in Broward and Palm Beach Counties, Florida. Annual data on financing costs for capital equipment and its replacement and savings in amounts of groundwater and associated water supply energy amounts and costs are also provided. The methods for determining and using the data presented in this article, along with related data, for the purpose of analyzing the feasibility of a widely-deployed residential rainwater harvesting (RWH) system in a heavily populated region in Southeast Florida are discussed in detail in Wurthmann [1]. The data presented here can be used by policy makers as a decision support tool for assessing broad outcomes related to population and single-family housing growth in Broward and Palm Beach Counties. They can also be used for more circumscribed outcomes related to developing implementation plans and assessing capital and financing costs and savings in water and energy amounts and costs for countywide RWH system installations. The data were derived from various expert projections, data from the U.S. Census Bureau, National Association of Home Builders (NAHB), Florida Department of Revenue and Property Tax, American Water Resources Association (AWRA), South Florida Water Management District (SFWMD), Electric Power Research Institute (EPRI), Energy Information Administration (EIA), and vendors of RWH system equipment, and customized calculations developed in Wurthmann [1].

**Table:** Specifications Table

Subject	Water Science and Technology
Specific subject area	Water, energy, and cost savings through regionally deployed residential rainwater harvesting systems for lawn irrigation
Type of data	Tables
How data were acquired	All data used in the analysis and presented here were derived from secondary datasets using calculations described herein. No software/programs or hardware were used.
Data format	All raw and analysed data is provided in the present data article.
Parameters for data collection	It is assumed that rates of water usage, sizes of homes and lots, numbers of people residing in homes, breakdowns of the percentages of homes based on numbers of bedrooms, and yearly ratios of numbers of single-family detached homes to numbers of people residing in Broward and Palm Beach Counties, Florida, as well as energy intensity of water and costs will remain constant through 2060.
Description of data collection	Data were collected from various secondary sources for further analysis and integration, as described in Wurthmann [1], to create the data tables contained in this paper.
Data source location	Data were collected for further analysis and integration, as described in Wurthmann [1], to create the data tables contained in this paper from the following secondary sources: high and low expert projections for populations at the turn of each decade from 2010 to 2060 [2,3]; population growth, averages of numbers of people residing in homes, and breakdowns of the percentages of homes based on numbers of bedrooms in Broward and Palm Beach Counties, Florida, for the years 2010 through 2017 [4]; rates of divergence between high and low population projections, based on length of time for the projections [5]; data on existing permits for pumping water from the Biscayne Aquifer and actual 2010 water supply pumpage quantities and corresponding figures for the populations using that water in Broward and Palm Beach Counties, Florida [2,6,7]; data on the average square footages of homes corresponding to numbers of bedrooms in the homes [8]; data on average lot sizes in Broward and Palm Beach Counties, Florida, in 2017 [9]; data on the required application rate of water to landscaped areas in Florida [10,11]; data on historical daily rainfall amounts from 2007 to 2017 in Fort Lauderdale, Florida [12]; vendor-based estimates of costs for RWH system equipment and its installation [13]; data on energy intensity of groundwater [14]; data on costs of energy for Florida industrial customers [15].
Data accessibility	All raw and analysed data are included with this article. Please see data in article and Table 1, Table 2, Table 3.
Related research article	Kurt Wurthmann Assessing storage requirements, water and energy savings, and costs associated with a residential rainwater harvesting system deployed across two counties in Southeast Florida Journal of Environmental Management Volume 252, 15 December 2019, 109673 [1]

**Table:** 

**Value of the Data** • These data are useful because they are the first to provide annual projections for Broward and Palm Beach Counties, Florida, during the period 2020–2060, for population growth, numbers of detached homes that must be fit with RWH system equipment each year so 100% of water demand by new residents is continuously offset, and costs and savings in groundwater and energy for its supply. • These data are useful because they summarize a unique integration of data using new analytic approaches, including techniques for RWH system sizing based on a nonparametric bootstrapping approach that synthetically generates daily precipitation, water supply, and irrigation demand using historical daily rainfall data. • These data can benefit researchers, analysts, and policy makers who have interests in outcomes related to population and single-family housing growth in Broward and Palm Beach Counties and developing implementation plans and assessing costs and savings in water and energy for countywide RWH system installations. • The methods used to derive these data can be modified for use in future studies of sustainable water management, which consider alternative contextual conditions, including changes in focal locations, population projections, percentages of populations residing in detached houses, per capita daily water usage, use of harvested rainwater, costs and energy usage, and climate.

## 1 Data

All raw and analysed data are included with this article. Please see data in the article and Table 1, Table 2, Table 3. Note, there can be increasing levels of uncertainty associated with population projections as the dates for the projections extend longer into the future [5]. To account for this uncertainty it is common practice to provide high and low series of population projections, rather than provide just one series of medium projections [2,3,5]. Accordingly, the present and related research articles [1,2,5] developed high and low projections for population growth in Broward and Palm Beach Counties.

**Table 1:** High projections for population growth and associated numbers of new and existing single-family homes to be fit or retrofit with RWH system equipment for lawn irrigation to continuously offset 100% of water demand created by new residents in such homes in Broward and Palm Beach Counties, in Florida.

Year	[A] High projections for Broward population	[B] High projections for Palm Beach population	[0.181* A] Broward County Number detached homes	[0.221*B] Palm Beach County Number detached homes	Number of new detached homes created and to be fit with RWH equipment each year by the growing population in Broward	Number of new detached homes created and to be fit with RWH equipment each year by the growing population in Palm Beach	Broward number of existing homes to be retrofit with RWH equipment each year to offset 100% of water demand created by new Broward & Palm Beach homes until all existing Broward homes retrofit	Palm Beach number of existing homes to be retrofit with RWH equipment each year to offset 100% of water demand created by new Broward & Palm Beach homes	Number of new and existing detached homes that must be fit or retrofit with RWH equipment each year in Broward	Number of new and existing detached homes that must be fit or retrofit with RWH equipment each year in Palm Beach
2020	1946700	1482900	352353	327721	0	0	0	0	0	0
2021	1966097	1498637	355864	331199	3511	3478	16161	0	19671	0
2022	1985687	1514541	359409	334713	3546	3515	16329	0	19874	0
2023	2005472	1530613	362990	338266	3581	3552	16498	0	20079	0
2024	2025454	1546856	366607	341855	3617	3590	16670	0	20287	0
2025	2045636	1563272	370260	345483	3653	3628	16843	0	20496	0
2026	2066018	1579862	373949	349149	3689	3666	17018	0	20707	0
2027	2086604	1596627	377675	352855	3726	3705	17195	0	20921	0
2028	2107395	1613571	381438	356599	3763	3745	17374	0	21137	0
2029	2128393	1630695	385239	360384	3801	3784	17554	0	21355	0
2030	2149600	1648000	389078	364208	3838	3824	17737	0	21575	0
2031	2168818	1661306	392556	367149	3478	2941	14455	0	17933	0
2032	2188208	1674719	396066	370113	3510	2964	14576	0	18086	0
2033	2207771	1688241	399607	373101	3541	2988	14699	0	18240	0
2034	2227509	1701872	403179	376114	3573	3012	14822	0	18395	0
2035	2247424	1715613	406784	379150	3605	3037	14947	0	18551	0
2036	2267517	1729465	410421	382212	3637	3061	15072	0	18709	0
2037	2287789	1743429	414090	385298	3669	3086	15199	0	18868	0
2038	2308243	1757505	417792	388409	3702	3111	15326	0	19028	0
2039	2328879	1771695	421527	391545	3735	3136	15455	0	19190	0
2040	2349700	1786000	425296	394706	3769	3161	15585	0	19354	0
2041	2370037	1808920	428977	399771	3681	5065	21316	0	24997	0
2042	2390549	1832134	432689	404902	3713	5130	11523	1008	15236	6138
2043	2411240	1855645	436434	410098	3745	5196	0	8139	3745	13335
2044	2432109	1879459	440212	415360	3777	5263	0	8229	3777	13492
2045	2453159	1903578	444022	420691	3810	5330	0	8320	3810	13650
2046	2474391	1928006	447865	426089	3843	5399	0	8411	3843	13810
2047	2495807	1952748	451741	431557	3876	5468	0	8504	3876	13972
2048	2517408	1977808	455651	437096	3910	5538	0	8598	3910	14136
2049	2539196	2003189	459595	442705	3944	5609	0	8693	3944	14302
2050	2561173	2028896	463572	448386	3978	5681	0	8789	3978	14470
2051	2577076	2058047	466451	454828	2878	6442	0	8419	2878	14862
2052	2593078	2087618	469347	461364	2896	6535	0	8518	2896	15053
2053	2609179	2117613	472261	467992	2914	6629	0	8618	2914	15247
2054	2625381	2148039	475194	474717	2932	6724	0	8719	2932	15443
2055	2641683	2178903	478145	481537	2951	6821	0	8822	2951	15643
2056	2658086	2210209	481114	488456	2969	6919	0	8926	2969	15845
2057	2674591	2241966	484101	495475	2987	7018	0	9031	2987	16049
2058	2691198	2274179	487107	502594	3006	7119	0	9138	3006	16257
2059	2707909	2306855	490131	509815	3025	7221	0	9246	3025	16467
2060	2724723	2340000	493175	517140	3043	7325	0	9355	3043	16680

**Table 2:** Expenses for RWH system capital equipment and its installation and replacement, the costs for financing these expenses,and savings in amounts of groundwater and associated water supply energy amounts and costs in Broward and Palm Beach Counties, in Florida.

Year	Total annual capital expenses for new installations and replacements of RWH equipment ($)	Total yearly payments for financing total yearly capital expenses ($)	Present values of total yearly payments for financing total yearly capital expenses ($)	Total amount of groundwater saved, each year, across both counties, by the RWH system (MGY)	Total amount of groundwater saved, each year, across both counties, by the RWH system (Mm³Y)	total annual water supply energy savings (kwh)	total annual water supply energy cost savings ($)	Present values of total annual energy cost savings ($)
2020	0	0	0	0	0.00	0	0	0
2021	98356783	8575193	8089804	1023	3.87	2147573	162786	153572
2022	99371541	17238856	15342521	2056	7.78	4317303	327252	291253
2023	100396778	25991905	21823305	3100	11.73	6509418	493414	414280
2024	101432603	34835261	27592790	4154	15.73	8724150	661291	523804
2025	102479125	43769859	32707384	5220	19.76	10961733	830899	620896
2026	103536455	52796638	37219547	6296	23.83	13222401	1002258	706552
2027	104604705	61916553	41178044	7384	27.95	15506395	1175385	781698
2028	105683988	71130565	44628196	8483	32.11	17813954	1350298	847193
2029	106774417	80439645	47612102	9593	36.31	20145322	1527015	903838
2030	107876108	89844776	50168854	10715	40.56	22500745	1705556	952374
2031	89667187	97662370	51447318	11647	44.09	24458585	1853961	976643
2032	90429590	105546434	52453344	12587	47.65	26433071	2003627	995741
2033	91198493	113497534	53212073	13535	51.24	28424347	2154565	1010144
2034	91973950	121516242	53746751	14492	54.86	30432553	2306788	1020294
2035	92756017	129603134	54078860	15456	58.51	32457837	2460304	1026599
2036	93544753	137758792	54228237	16429	62.19	34500341	2615126	1029435
2037	94340212	145983802	54213190	17410	65.90	36560214	2771264	1029149
2038	95142453	154278754	54050604	18399	69.65	38637604	2928730	1026063
2039	95951534	162644246	53756039	19397	73.42	40732660	3087536	1020471
2040	96767513	171080879	53343827	20403	77.23	42845532	3247691	1012646
2041	223341345	181977603	53529695	21702	82.15	45574510	3454548	1016174
2042	236931467	193970704	53827859	23016	87.13	48334257	3663737	1016706
2043	252475301	207229603	54252142	24345	92.16	51125136	3875285	1014539
2044	255236242	220638905	54493076	25689	97.24	53947474	4089219	1009949
2045	258028059	234200370	54568365	27048	102.39	56801637	4305564	1003191
2046	260851104	247915778	54494374	28423	107.59	59687995	4524350	994498
2047	263705733	261786930	54286220	29813	112.85	62606922	4745605	984086
2048	266592308	275815651	53957855	31218	118.17	65558799	4969357	972156
2049	269511193	290003785	53522153	32640	123.56	68544008	5195636	958890
2050	272462758	304353199	52990975	34078	129.00	71562939	5424471	944455
2051	252677256	318565159	52325869	35491	134.35	74531876	5649516	927960
2052	255441830	332951678	51593325	36922	139.77	77536931	5877299	910731
2053	258239561	347515080	50801917	38371	145.25	80578583	6107857	892884
2054	261070872	362257720	49959517	39837	150.80	83657318	6341225	874528
2055	263936196	377181988	49073345	41321	156.42	86773627	6577441	855759
2056	266835969	392290306	48150015	42823	162.10	89928007	6816543	836668
2057	269770634	407585130	47195579	44343	167.86	93120966	7058569	817334
2058	272740640	423068949	46215565	45882	173.68	96353014	7303558	797832
2059	275746441	438744288	45215020	47440	179.58	99624673	7551550	778229
2060	278788499	454613707	44198539	49017	185.55	102936467	7802584	758584

**Table 3:** Low projections for population growth and associated amounts of total water demand in Broward and Palm Beach Counties, in Florida.

Year	Low projections for Broward population	Low projections for Palm Beach population	Broward total water demand (MGD)	Palm Beach total water demand (MGD)	Total water demand Broward + Palm Beach (MGD)	Broward total water demand (Mm³D)	Palm Beach total water demand (Mm³D)	Total water demand Broward + Palm Beach (Mm³D)
2020	1946700	1482900	255	251	506	0.97	0.95	1.92
2021	1944351	1488154	255	252	507	0.96	0.96	1.92
2022	1942005	1493426	254	253	508	0.96	0.96	1.92
2023	1939662	1498718	254	254	508	0.96	0.96	1.92
2024	1937322	1504027	254	255	509	0.96	0.97	1.93
2025	1934985	1509356	253	256	509	0.96	0.97	1.93
2026	1932650	1514704	253	257	510	0.96	0.97	1.93
2027	1930318	1520070	253	258	511	0.96	0.98	1.93
2028	1927989	1525456	253	259	511	0.96	0.98	1.93
2029	1925663	1530861	252	260	512	0.95	0.98	1.94
2030	1923340	1536284	252	260	512	0.95	0.99	1.94
2031	1919066	1538725	251	261	512	0.95	0.99	1.94
2032	1914801	1541169	251	261	512	0.95	0.99	1.94
2033	1910547	1543618	250	262	512	0.95	0.99	1.94
2034	1906301	1546070	250	262	512	0.95	0.99	1.94
2035	1902065	1548526	249	263	512	0.94	0.99	1.94
2036	1897839	1550986	249	263	512	0.94	1.00	1.94
2037	1893621	1553450	248	263	511	0.94	1.00	1.94
2038	1889414	1555918	247	264	511	0.94	1.00	1.94
2039	1885215	1558389	247	264	511	0.93	1.00	1.93
2040	1881026	1560865	246	265	511	0.93	1.00	1.93
2041	1874725	1558666	246	264	510	0.93	1.00	1.93
2042	1868444	1556470	245	264	509	0.93	1.00	1.93
2043	1862185	1554277	244	264	507	0.92	1.00	1.92
2044	1855946	1552087	243	263	506	0.92	1.00	1.92
2045	1849729	1549900	242	263	505	0.92	0.99	1.91
2046	1843532	1547717	241	262	504	0.91	0.99	1.91
2047	1837356	1545536	241	262	503	0.91	0.99	1.90
2048	1831201	1543359	240	262	502	0.91	0.99	1.90
2049	1825066	1541184	239	261	500	0.90	0.99	1.89
2050	1818952	1539013	238	261	499	0.90	0.99	1.89
2051	1812859	1539013	237	261	498	0.90	0.99	1.89
2052	1806786	1539013	237	261	498	0.90	0.99	1.88
2053	1800733	1539013	236	261	497	0.89	0.99	1.88
2054	1794700	1539013	235	261	496	0.89	0.99	1.88
2055	1788688	1539013	234	261	495	0.89	0.99	1.87
2056	1782696	1539013	233	261	494	0.88	0.99	1.87
2057	1776723	1539013	233	261	494	0.88	0.99	1.87
2058	1770771	1539013	232	261	493	0.88	0.99	1.87
2059	1764839	1539013	231	261	492	0.87	0.99	1.86
2060	1758927	1539013	230	261	491	0.87	0.99	1.86

Table 1 presents annual data on high projections for population growth and associated numbers of new and existing single-family detached homes to be fit or retrofit with RWH system equipment for lawn irrigation in order to continuously offset 100% of water demand created by new residents in such homes in Broward and Palm Beach Counties, in Florida. Column 1 shows the years of data, which extend from 2020 to 2060. Columns 2–5 show the high population projections for numbers of people and single-family detached homes in Broward and Palm Beach Counties, respectively. Columns 6 and 7 show the numbers of new single-family detached homes that are created and must be fit with RWH equipment each year due to the growing populations in Broward and Palm Beach Counties, respectively. Column 8 shows the numbers of existing single-family detached homes that must be retrofit with RWH equipment each year in Broward County, in order to offset 100% of the water demand created that year by newly built Broward and Palm Beach County single-family detached homes. The process of retrofitting Broward County homes with RWH system equipment continues until all existing Broward single-family detached homes are so retrofit. At that point, the process of fitting new and retrofitting existing Palm Beach County single-family detached homes with RWH system equipment begins. This sequence is chosen because it is more expensive to fit Palm Beach versus Broward County homes with RWH system equipment. Column 9 shows the numbers of existing single-family detached homes that must be retrofit with RWH equipment each year in Palm Beach County, in order to offset 100% of the water demand created that year by newly built Broward and Palm Beach County single-family detached homes. The process of retrofitting existing Palm Beach County single-family detached homes with RWH system equipment continues through 2060. Columns 10 and 11 show the total numbers of new and existing single-family detached homes that must be fit or retrofit with RWH equipment each year in Broward (sum of columns 6 and 8) and Palm Beach (sum of columns 7 and 9) Counties, respectively.

Table 2 presents annual data on expenses for RWH system capital equipment and its installation and replacement, the costs for financing of these expenses, and savings in amounts of groundwater and associated water supply energy amounts and costs in Broward and Palm Beach Counties, in Florida. Column 1 shows the years of data, which extend from 2020 to 2060. Column 2 shows the total expenses required in each year for RWH system capital equipment and its installation and replacement. Column 3 shows the total dollar amount spent each year for financing the expenses over a 20-year period, at an interest rate of six percent, structured with level debt service. Column 4 shows the present values of the annual financing costs, using a discount rate of six percent. Columns 5 and 6 show the total amount of groundwater saved, each year, across both counties, by the RWH system in millions of gallons per year (MGY) and millions of cubic meters per year (Mm³Y), respectively. Column 7 shows the total annual water supply energy savings in kilowatt hours (kwh). Column 8 shows the total dollar amount of water supply energy saved each year. Column 9 shows the present values of the annual dollar amounts of water supply energy savings, using a discount rate of six percent.

Table 3 presents annual data on low projections for population growth and associated amounts of total water demand in Broward and Palm Beach Counties, in Florida. Column 1 shows the years of data, which extend from 2020 to 2060. Columns 2–5 show the low population projections for numbers of people and the corresponding amounts of total water demand in millions of gallons per day in Broward and Palm Beach Counties, respectively. Column 6 shows the combined amount of total water demand in millions of gallons per day in both Broward and Palm Beach Counties, together. Columns 7 and 8 show the amounts of total water demand in millions of cubic meters per day in Broward and Palm Beach Counties, respectively. Column 9 shows the combined amount of total water demand in millions of cubic meters per day in both Broward and Palm Beach Counties, together.

Note, some water management projects are designed to meet the needs of just one county, while others are designed to meet the needs of multiple counties [1,2,6]. Thus, it can be useful to consider water demands at the level of the individual county, as presented in columns 4, 5, 7, and 8, as well as across both counties, as presented in columns 6 and 9. Broward and Palm Beach Counties are adjacent and there are significant links between the water supply systems in these counties. For example, recent reservoir-based water management systems have been designed to simultaneously meet the growing water supply needs of both Broward and Palm Beach Counties [1,2,6].

## 2 Experimental design, materials, and methods

The data presented Table 1, Table 2, Table 3 in the present article were acquired and analysed as described in Wurthmann [1]. This section of the present article provides additional details concerning how the data shown in Table 1, Table 2, Table 3 were acquired and analysed.

The data shown in Table 1, Table 2, Table 3 are based on assumptions that the following items remain constant: 1. rates of water usage; 2. sizes of homes and lots; 3. numbers of people residing in homes; 4. breakdowns of the percentages of homes based on numbers of bedrooms; and 5. yearly ratios of numbers of single-family detached homes to numbers of people residing in Broward and Palm Beach Counties, Florida. It is also assumed that the energy intensity of water and costs will remain constant through 2060. These assumptions may not hold if there are changes in individual and societal perceptions and norms, or technological, political, legal, economic, and environmental factors. Accordingly, analysts may wish to consider the sensitivity of some of the data in the present article to potential changes in the underlying assumptions. For example, given the long-term nature of the data and particular susceptibility of the region to the effects of climate change, analysts might wish to consider how the data provided herein could be affected by outcomes including: 1. more heavy and sporadic rainfall events; 2. more intense drought-flood cycles; 3. higher average temperatures; 4. significant changes in perceptions, behaviors, regulations, and property values; and 5. potentially large-scale climate migration [1].

In Table 1, turn-of-decade values for populations shown in columns 2 and 3 were collected from secondary sources as raw data [2,3]. These turn-of-decade values were then used as the endpoints for calculating compound annual growth rates (CAGRs) to fill in population levels for each year, during each decade. The average ratios of numbers of detached houses to numbers of people, for the years 2010 through 2017, for Broward and Palm Beach Counties, were calculated from Census data as 0.181 and 0.221, respectively [1,4]. These ratios were multiplied by the population data in columns 2 and 3 to determine the numbers of detached homes in Broward and Palm Beach Counties shown in columns 4 and 5, respectively. Numbers of new detached homes created and to be fit with RWH equipment each year are shown in columns 6 and 7. These data were calculated as the year-to-year changes in the numbers of detached homes in Broward and Palm Beach Counties shown in columns 4 and 5, respectively.

The data shown in columns 8 and 9 in Table 1 were determined based on the following considerations. As per Wurthmann [1], meeting 100% of the outdoor irrigation needs of 142.43 gallons per day at single-family detached homes in Broward County only meets 39.2% of the total water needs at such homes. Note total household water needs were the product of the average number of residents per household, based on 2017 US census data [4], and per capita water usage data [1,2,6,7]. As per Wurthmann [1], per capita water usage was determined “by dividing actual 2010 water supply pumpage quantities by the corresponding figures for the populations using that water in Broward and Palm Beach Counties” [1]. The outdoor irrigation needs were calculated assuming an average area of yard in Broward County requiring irrigation of 3198.78 square feet and an average required amount of irrigation of 0.5 inches, once per week, year round [1,[9], [10], [11]]. Note, the average area of yard requiring irrigation was calculated as 50% of the difference between the average lot size and home square footage for the county of interest [1]. The average home square footage (and rooftop area) values for detached homes in Broward and Palm Beach Counties were determined to be 2223.45 and 2284.80 square feet, respectively [1]. These values were determined by multiplying the percentages of homes in the counties of interest, based on numbers of bedrooms [4], by data on the average square footage of homes for sizes corresponding to number of bedrooms [8]. Thus, for each newly created single-family detached household in Broward County that is equipped for RWH, 1.55 existing single-family detached homes in Broward County must also be so equipped in order to offset the additional total water demand.

Similarly, meeting 100% of the outdoor irrigation needs of 233.04 gallons per day at single-family detached homes in Palm Beach County only meets 53.1% of the total water needs at such homes [1]. The outdoor irrigation needs were calculated assuming an average area of yard in Palm Beach county requiring irrigation of 5233.60 square feet and an average required amount of irrigation of 0.5 inches, once per week, year round [1,[9], [10], [11]]. Thus, for each newly created single-family detached household in Palm Beach County that is equipped for RWH, 0.88 existing single-family detached homes in Palm Beach County must also be so equipped in order to offset the additional total water demand. Further, since single-family detached homes in Palm Beach versus Broward County use more water in total and for outdoor irrigation, appropriate conversion factors were applied to determine equivalence between numbers of single-family detached households and water usage across the two counties.

The cost figures shown in Table 2 are based on the assumptions that the cost per household for installed RWH system equipment is $5000 and $10000 in Broward and Palm Beach Counties, respectively [1], the energy intensity of water is 2100 kwh/MG [14], and the cost of energy is $0.0758 per kwh [15]. The cost per household for installed RWH system equipment is based on the assumption that each household in Broward and Palm Beach Counties, respectively, is to be equipped with four and eight 2000-gallon, above-ground, plastic cisterns [1]. These equipment requirements were determined using a nonparametric bootstrapping method for synthetically generating daily precipitation, water supply, and irrigation demand for rainwater harvesting system storage sizing [1,16]. The cost components, on a per cistern basis, included $800 for the cistern, $250 for all associated equipment (conveyance, filtration, disinfection, etc.), and $200 for installation labor [1,13]. Note, it is assumed that the costs of including RWH system equipment on new and existing homes is the same.

The data in column 2 in Table 2 were determined by multiplying the cost per household for installed RWH system equipment by the number of new and existing detached homes that must be fit or retrofit with RWH equipment each year from Table 1. The data in column 3 in Table 2 were determined by summing all required, annual level payment amounts for each year, in the period 2020 through 2060. It is assumed all annual total capital outlays required for the installation and replacement of RWH equipment are financed using 20-year schedules of level payments. The values in these schedules of level payments were determined by multiplying each annual capital outlay by the inverse of the annuity factor with an interest rate of six percent. Note, “it is assumed that all installed RWH system equipment must be replaced, at its initial installation cost, every 20 years” [1].

The data in column 4 in Table 2 were determined by multiplying the data in column 3 by a present value factor corresponding to a six percent interest rate. The data in columns 5 and 6 in Table 2 were determined by multiplying the total number of new houses fit and existing houses retrofit with RWH system equipment in Broward and Palm Beach Counties by the amounts of water used for landscape irrigation by households in those counties [1]. The data in column 7 in Table 2 were determined by multiplying the data in column 5 by a groundwater energy intensity value of 2100 kwh/MG [14]. The data in column 8 in Table 2 were determined by multiplying the data in column 7 by a cost of energy for Florida industrial customers of $0.0758 per kwh [15]. The data in column 9 in Table 2 were determined by multiplying the data in column 8 by a present value factor corresponding to a six percent interest rate.

In Table 3, the values for populations shown in columns 2 and 3 were determined as follows. The 2020 population projections for both Broward and Palm Beach Counties were the same as those shown in Table 1 (i.e. The populations in the two counties in the present time are assumed to be the same). From this common starting population level, the rate of change projections for low population growth, for future decades in Broward County are assumed to result in percentage changes in population “of −1.2% from 2020 to 2030, -2.2% from 2030 to 2040, and −3.3% for each of the remaining two decades” [1]. The rate of change projections for low population growth, for future decades in Palm Beach County are assumed to result in percentage changes in population “of 3.6% from 2020 to 2030, 1.6% from 2030 to 2040, −1.4% from 2040 to 2050, and 0% for 2050–2060” [1]. The turn-of-decade values were then used as the endpoints for calculating CAGRs to fill in population levels for each year, during each decade, for both Broward and Palm Beach Counties. The water demand data shown in the remaining columns in Table 3 were determine by multiplying population data by total daily per capita water usage. It is assumed total daily per capita water usage remains constant at 130.97 and 169.56 gallons in Broward and Palm Beach Counties, respectively [1].

Additional information related to the materials, methods, and assumptions used to integrate data from multiple secondary sources to create the data presented Table 1, Table 2, Table 3 in the present paper is provided in Wurthmann [1].
